# Circulating Calprotectin Distinguishes Metastatic Melanoma and Predicts Liver Metastasis

**DOI:** 10.3390/ijms26168028

**Published:** 2025-08-20

**Authors:** István Szász, Viktória Koroknai, Tünde Várvölgyi, Gabriella Emri, Imre Lőrinc Szabó, Margit Balázs

**Affiliations:** 1 HUN-REN-UD Public Health Research Group, Department of Public Health and Epidemiology, Faculty of Medicine, University of Debrecen, 4028 Debrecen, Hungary; 2 Department of Public Health and Epidemiology, Faculty of Medicine, University of Debrecen, 4028 Debrecen, Hungary; 3 Department of Dermatology, Faculty of Medicine, University of Debrecen, 4028 Debrecen, Hungary

**Keywords:** melanoma, organ specific metastasis, liver metastasis, calprotectin, liquid biopsy, S100B and LDH, biomarker

## Abstract

Calprotectin, a heterodimer of the S100A8 and S100A9 proteins, has been implicated in cancer-related inflammation and metastasis. Its role in melanoma progression, particularly in organ-specific metastasis, remains underexplored. In this retrospective study, plasma calprotectin levels were measured in 201 individuals, including healthy controls (*n* = 22), melanoma patients without evidence of metastasis (*n* = 71), and patients with metastatic melanoma (*n* = 108). Calprotectin concentrations were determined using the ELISA assay. Receiver operating characteristic (ROC) curve analyses were used to evaluate its diagnostic value, both alone and in combination with established biomarkers S100B and LDH. Plasma calprotectin levels were significantly elevated in patients with metastatic melanoma compared to non-metastatic patients (*p* < 0.001). Calprotectin showed moderate diagnostic value (AUC = 0.672), which improved to 0.755 when combined with S100B and LDH. Organ-specific analysis revealed that patients with liver metastases exhibited the highest calprotectin concentrations, with good discriminatory power (AUC = 0.710). No significant association was found between calprotectin levels and the type of metastasis identified (lymphatic vs. hematogenous). Logistic regression analysis showed that calprotectin levels above 2728 ng/mL were associated with a 7.4-fold increased risk of liver metastasis. Calprotectin is a promising blood-based biomarker that may enhance the detection of metastatic melanoma, particularly in cases with liver involvement. These findings suggest that calprotectin could be integrated into multivariable prediction models to improve risk stratification in clinical practice.

## 1. Introduction

It is well recognized that the development and progression of tumors, including melanoma, are strongly influenced by the tumor microenvironment. A dynamic, bidirectional interaction exists between tumor cells and surrounding stromal and immune cells. This crosstalk, mediated through altered receptor expression patterns and the secretion of soluble factors, plays a critical role in modulating tumor progression [1]. Among the molecular players involved in this tumor–host cell interaction, S100 proteins have emerged as key regulators. The S100 family comprises 27 calcium-binding proteins, which are named for their solubility in 100% saturated ammonium sulfate at neutral pH. These proteins are involved in diverse biological processes such as cell proliferation, apoptosis, calcium homeostasis, migration, invasion, and inflammation. Due to their extracellular release during tissue damage or stress, many S100 proteins function as Damage-Associated Molecular Pattern (DAMP) molecules, also referred to as alarmins, which serve as an alarm to the immune system [2,3,4,5]. In melanoma, the most widely studied member of this family is S100B. This protein is highly expressed in melanoma cells and is used clinically as a marker of tumor progression, facilitating disease monitoring, assessment of recurrence, and therapeutic response. Elevated serum levels of S100B have been correlated with advanced disease stages and poorer prognosis [6,7,8].

Other S100 proteins with importance in melanoma include S100A8 and S100A9, which form heterodimers commonly known as calprotectin (also referred to as leucocyte L1 protein, calgranulin A/B, or the Myeloid-Related Protein (MRP8/14)). Unlike S100B, calprotectin is not synthesized by melanoma cells themselves but is predominantly produced by neutrophils, monocytes, and keratinocytes within the tumor microenvironment, particularly under conditions of cellular stress or inflammation [9]. Notably, melanoma cells can express receptors for calprotectin, including RAGE (Receptor for Advanced Glycation End-Products), EMMPRIN (Extracellular Matrix Metalloproteinase Inducer, also known as Basigin or CD147), TLR4 (Toll-like receptor 4), and MCAM (Melanoma Cell Adhesion Molecule, CD146) [10,11,12,13]. Calprotectin also exerts chemotactic effects, promoting the direct migration of melanoma cells toward calprotectin-rich sites. This may facilitate the establishment of pre-metastatic niches and contribute to metastatic spread, particularly in the lungs. These observations are consistent with the classical “seed and soil” hypothesis of metastasis [14,15,16].

In this study, we aimed to explore the relationship between circulating calprotectin levels and clinicopathological parameters in melanoma patients, with a particular focus on metastatic status and organ-specific dissemination. Our findings demonstrate that plasma calprotectin levels are significantly elevated in patients with metastatic melanoma and may help distinguish individuals with liver metastasis. These results support the potential utility of calprotectin as a complementary biomarker in multivariable predictive models to improve metastatic detection and risk stratification.

## 2. Results

### 2.1. Plasma Calprotectin Levels Are Elevated in Metastatic Melanoma

A total of 201 plasma samples were analyzed in this study, categorized into three groups. The first group, comprising healthy individuals, included 22 participants and served as the control group. The second group consisted of 71 melanoma patients without clinical evidence of metastasis at the time of sampling; these individuals were classified as tumor-free based on negative CT imaging performed at least one month prior to blood collection. The third group included 108 patients with confirmed metastatic melanoma at the time of sampling.

**Figure 1:**
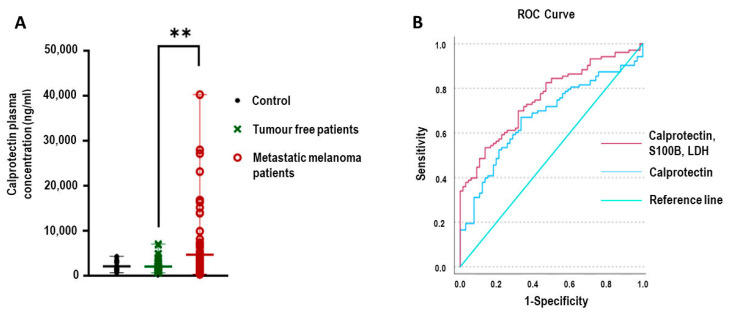
Comparison of plasma calprotectin levels across study groups and their diagnostic value in detecting metastatic melanoma. (**A**) Plasma calprotectin concentrations in the control group (*n* = 22, black), tumor-free melanoma group (*n* = 71, green), and metastatic melanoma group (*n* = 108, red). Statistical significance was assessed with Bonferroni correction for multiple comparisons. ** *p* < 0.01. (**B**) Receiver operating characteristic (ROC) curves for plasma calprotectin alone and for the combined biomarker model (calprotectin, S100B, and LDH), showing improved discriminatory performance for metastatic melanoma detection.

Plasma calprotectin levels were measured across all three groups. As shown in Figure 1A, calprotectin concentrations were significantly elevated in patients with metastatic melanoma compared to tumor-free patients (*p* = 0.001). To assess the diagnostic utility of calprotectin, receiver operating characteristic (ROC) curve analysis was performed. The AUC for calprotectin alone was 0.672 (95% CI: 0.591–0.753). When calprotectin was combined with established melanoma biomarkers S100B and lactate dehydrogenase (LDH), the model’s diagnostic performance improved, with a combined AUC of 0.755 (95% CI: 0.684–0.827), as illustrated in Figure 1B.

### 2.2. Plasma Calprotectin Levels Vary by Site of Metastasis

We investigated the relationship between plasma calprotectin levels and a range of clinicopathological parameters in patients with melanoma, as summarized in Table 1. Consistent with prior observations, plasma calprotectin concentrations were significantly elevated in patients with metastasis compared to those without evidence of metastasis (*p* < 0.001). This finding supports the hypothesis that calprotectin levels may reflect alterations associated with melanoma progression.

To further explore this relationship, we categorized patients within the metastatic cohort according to the predominant route of metastatic dissemination—hematogenous versus lymphatic spread. Interestingly, no statistically significant difference in calprotectin concentrations was observed between these two groups, suggesting that metastasis progression may play a more critical role in modulating calprotectin levels than the specific route of dissemination.

We next performed an organ-specific analysis within the group of patients with confirmed metastatic disease. Among patients with single-organ involvement, those with hepatic metastases exhibited remarkably higher plasma calprotectin levels compared to individuals with brain or pulmonary metastases. This elevation may reflect the liver’s central role in systemic inflammation and immune modulation, as well as its high vascularization and direct exposure to circulating inflammatory mediators.

The highest plasma calprotectin levels were detected in patients with metastatic involvement from multiple organs, suggesting a possible additive effect of widespread tumor progression and systemic inflammation. These results suggest that these findings underscore the potential of plasma calprotectin as a surrogate biomarker of metastatic load and organ-specific involvement, with particular relevance to hepatic and multi-organ metastasis.

**Figure 2:**
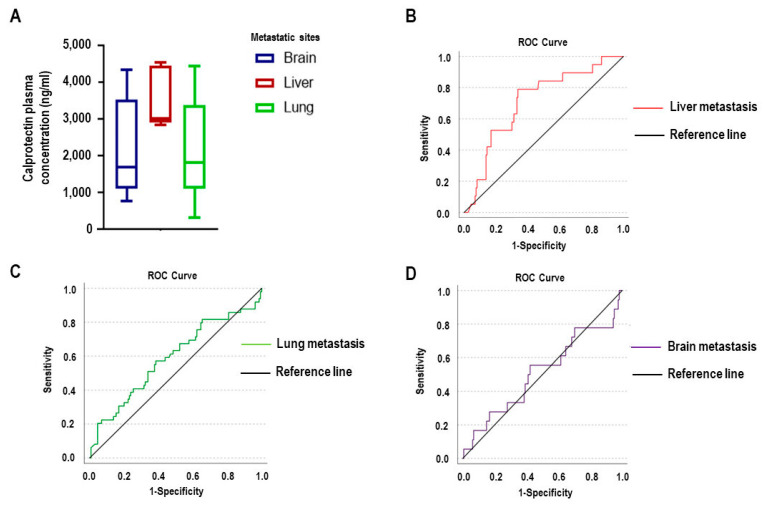
Diagnostic value of plasma calprotectin for liver metastases by metastatic site. (**A**) Plasma calprotectin concentrations in patients with brain (*n* = 5, lilac box), liver (*n* = 5, red box), and lung (*n* = 13, green box) metastases. (**B**) ROC curve for the association between plasma calprotectin levels and liver metastasis (red line; AUC = 0.710, 95% CI: 0.594–0.825). (**C**) ROC curve for the association between calprotectin levels and lung metastasis (green line; AUC = 0.589, 95% CI: 0.490–0.687). (**D**) The ROC curve for the association between calprotectin levels and brain metastasis (lilac line; AUC = 0.509, 95% CI: 0.353–0.665).

Among patients with multi-organ involvement (*n* = 51), liver metastases were present in 19 cases, lung metastases were present in 49 cases, and brain metastases were present in 18 cases. ROC curve analysis showed that calprotectin had good discriminatory ability for detecting liver metastasis (AUC = 0.710, 95% CI: 0.594–0.825) but performed poorly regarding lung (AUC = 0.589, 95% CI: 0.490–0.687) and brain metastases (AUC = 0.509, 95% CI: 0.353–0.665) (Figure 2). In cases with liver metastasis, we tested S100B and LDH, but we did not find a correlation. (AUC: 0.684, CI: 0.556–0.812; AUC: 0.506, CI: 0.343–0.670, respectively).

To further examine the prognostic value of calprotectin in liver metastasis, we calculated an optimal cut-off value using the Youden index (2728.2 ng/mL). Logistic regression analysis revealed that patients with calprotectin levels above this threshold had a 7.4-fold increased risk of harboring liver metastases compared to those below the threshold (*p* < 0.001; OR: 7.361, 95% CI: 2.329–23.261). These findings indicate that elevated plasma calprotectin may serve not only as a general marker of metastatic disease in melanoma but also as a specific indicator of hepatic metastatic involvement.

## 3. Discussion

Calprotectin, a heterodimer composed of the S100A8 and S100A9 proteins, has been increasingly recognized as a key player in tumor-associated inflammation. A growing body of evidence suggests that calprotectin is highly expressed in several types of cancer and contributes to tumor progression, immune modulation, and metastasis, particularly in inflammation-associated malignancies [17]. Moreover, its role in organ-specific metastasis formation is gaining attention, supporting Paget’s classical “seed and soil” hypothesis, which posits that metastasis is a non-random process shaped by interactions between cancer cells and the microenvironment of specific organs [18].

In this study, we aimed to explore the relationship between circulating calprotectin levels and clinicopathological parameters in melanoma patients, with a particular focus on metastatic status and organ-specific dissemination. Previous findings, particularly those reported by Wagner and colleagues, highlighted that calprotectin expression is elevated in metastatic melanoma tissues as well as in the serum of patients with advanced-stage disease. Their work demonstrated a strong correlation between high calprotectin levels and poor prognosis in stage III and IV melanoma, suggesting that calprotectin may serve both as a biomarker and as a functional mediator of melanoma progression [19].

Our results are in line with and extend these previous findings. We found that plasma calprotectin levels were significantly higher in metastatic melanoma patients compared to those without detectable metastases. Moreover, when calprotectin was combined with established melanoma biomarkers S100B and LDH, the diagnostic accuracy for identifying metastatic disease improved substantially. Specifically, the area under the ROC curve (AUC) increased from 0.672 (calprotectin alone) to 0.755 when used in a combined biomarker model, supporting its additive value in melanoma monitoring.

Interestingly, while calprotectin levels did not differ based on the assumed route of metastatic spread (lymphatic vs. hematogenous), a clear difference emerged when distant metastases were stratified by organ involvement. Patients with liver metastases had markedly higher plasma calprotectin concentrations compared to those with brain or lung metastases. Although the number of cases in each organ-specific subgroup was limited, the ROC analysis showed that calprotectin could reliably distinguish liver metastasis (AUC = 0.710) but had poor discriminative power for lung (AUC = 0.589) and brain metastases (AUC = 0.509).

The liver-specific elevation of calprotectin levels may reflect multiple, not commonly exclusive, biological mechanisms. First, it is possible that patients with liver metastases had greater metastatic involvement, leading to a stronger systemic inflammatory response and increased release of calprotectin. Alternatively, the liver microenvironment itself may selectively induce calprotectin expression through tissue-specific inflammatory pathways or through crosstalk with melanoma cells. These possibilities remain speculative and highlight a limitation of our study: while we observed a strong association between liver metastases and elevated calprotectin levels, we could not directly assess tumor progression or local tissue expression.

Other studies have proposed that calprotectin acts as a chemoattractant and contributes to the formation of pre-metastatic niches. For example, murine models from Arjun et al. demonstrated the presence of an S100A8/A9 concentration gradient that directed melanoma cells toward the lungs, where the highest concentrations of calprotectin were found [15]. While our data do not support a similar lung-specific role in human samples, the observation of elevated systemic calprotectin in patients with liver involvement may point to a parallel mechanism or to organ-specific variations in the inflammatory milieu that favor hepatic colonization.

It is also important to acknowledge the limitations of our study. The sample size for organ-specific subgroups—particularly liver and brain metastases—was relatively small, which limits the statistical power and generalizability of our findings. Furthermore, we did not perform tissue-level validation of calprotectin expression in metastatic sites, nor did we quantify the overall extent of metastatic disease, which may confound the interpretation of circulating calprotectin levels. Despite these limitations, our results add new insight into the potential role of calprotectin in melanoma dissemination and may serve as a basis for future studies.

In conclusion, our findings highlight calprotectin as a promising circulating biomarker associated with metastatic melanoma and point to a potential organ-specific link with liver metastases. To validate these results and better understand the biological role of calprotectin in melanoma dissemination, future studies should incorporate larger patient cohorts, mechanistic investigations, and tissue-level analyses.

## 4. Materials and Methods

### 4.1. Melanoma Patients and Tumor Samples

A total of 179 patients diagnosed with melanoma were retrospectively enrolled in this study between 4 April 2019 and 22 September 2022. Clinical and laboratory data were retrieved from the institutional electronic medical records (MedSolution and UDMED systems) at the University of Debrecen (Supplementary Table S1). The study protocol received ethical approval from the Ethics Committee of the Hungarian Medical Research Council (approval numbers: TUKEB 17876–2018/EKU and IV/1711-4/2021/EKU).

For each patient, relevant clinical parameters were recorded, including age, sex, histological subtype of the primary melanoma, tumor thickness (Breslow thickness), presence of ulceration, anatomical location, Clark level of invasion, and pathological T stage based on the 8th edition of the American Joint Committee on Cancer (AJCC) TNM classification. Laboratory values for serum S100B and lactate dehydrogenase (LDH) were also documented. Peripheral blood was collected from 179 melanoma patients, including 71 individuals without evidence of metastasis and 108 with confirmed metastatic disease. Serum levels of S100B (measured via chemiluminescent immunoassay, LIAISON^®^ S100, Madison, WI, USA) and LDH (determined using an automated colorimetric assay) were routinely assessed during follow-up, typically at three-month intervals.

Metastatic status was established using PET-CT, CT imaging, or soft tissue ultrasound performed within three months of blood being drawn. The metastatic cohort consisted of (1) patients with newly diagnosed metastases who had not yet begun systemic therapy and (2) patients already receiving targeted therapy (BRAF and MEK inhibitors) or immune checkpoint inhibitors. The non-metastatic group comprised (1) patients with no evidence of disease following primary tumor excision, (2) individuals in complete remission following systemic therapy, and (3) patients rendered metastasis-free after lymph node dissection. 

### 4.2. Measurement of Plasma Calprotectin Levels

Plasma calprotectin concentrations were determined using a commercially available human calprotectin sandwich ELISA kit (Assay Genie, Cat. No. HUFI03073; 25 Windsor Place, Dublin 2, D02 VY42, Ireland). The assay range of this kit is 0.625–40 ng/mL, with a reported sensitivity of 0.375 ng/mL. All measurements were performed in accordance with the manufacturer’s protocol. Based on preliminary optimization experiments, a 1:120 dilution was applied to all plasma samples. When the calprotectin concentration exceeded the dynamic range of the assay at this dilution, further dilutions of 1:200, 1:400, or 1:800 were applied as necessary. Samples that produced identical absorbance values across serial dilutions, indicating saturation or matrix interference, were excluded from the analysis.

To assess the potential effect of repeated freeze–thaw cycles on calprotectin stability, selected plasma samples underwent 0, 5, and 10 freeze–thaw cycles. No significant variation in calprotectin concentrations was observed, indicating stability under these conditions. Absorbance was measured at 450 nm using an Epoch™ Microplate Spectrophotometer (BioTek Instruments, Winooski, VT, USA).

### 4.3. Statistical Analysis

Statistical analyses were carried out using IBM SPSS Statistics 26.0 software (IBM Company, Palo Alto, CA, USA). The normality of the data was assessed using the Shapiro–Wilk test. The Kruskal–Wallis test was used to compare the plasma calprotectin levels in melanoma patients with different outcomes and the control group. Significance values were adjusted using the Bonferroni correction for multiple tests. The ROC curve and AUC (area under the curve) were calculated to assess the predictive performance of plasma calprotectin levels. The combined AUC of calprotectin, S100B, and LDH was estimated using the predicted probability value calculated via binary logistic regression analyses. The prognostic value of calprotectin in liver metastasis was calculated using an optimal cut-off value determined by the Youden index. Logistic regression analysis was used to assess the associations between calprotectin levels and the presence of liver metastasis. *p* < 0.05 was considered statistically significant.

## Figures and Tables

**Table 1:** Plasma calprotectin concentration and clinicopathological characteristics of melanoma patients.

Parameter	No. of Plasma Samples (%)	Calprotectin (ng/mL) (Mean ± SD)	*p* Value ^1^
Gender			0.931
Female	79 (44.1)	3493.8 ± 451.9	
Male	100 (55.9)	3779.7 ± 596.1	
Age			0.615
<65 years	88 (49.2)	3771.0 ± 491.8	
>65 years	91 (50.8)	3560.5 ± 604.1	
**Metastasis**			**<0.001**
Absent	71 (39.7)	2076.0 ± 159.6	
Present	108 (60.3)	4690.5 ± 614.3	
Metastasis type			0.626
Hematogenous ^2^	32 (17.9)	4103.6 ± 854.9	
Lymphatic ^3^	76 (42.5)	4937.6 ± 796.9	
Therapy (metastatic patients)			0.334
None	33 (18.4)	4426.4 ± 875.4	
Target	20 (11.2)	6431.9 ± 2074.2	
Immune	55 (30.7)	4215.7 ± 787.4	
Type of distant organ metastasis			0.092
Brain	5 (2.8)	2199.6 ± 621.7	
Liver	5 (2.8)	3555.1 ± 373.6	
Lung	13 (7.3)	2108.8 ± 366.1	
Multiple organs	51 (28.5)	5009.6 ± 816.6	

^1^ Mann–Whitney Wilcoxon test; ^2^ distant and/or cutaneous and lymph node metastasis; ^3^ only lymph node metastasis.

## Data Availability

The data used to support the findings of this study are available from the corresponding author upon request.

## Supplementary Materials

The following supporting information can be downloaded at https://www.mdpi.com/article/10.3390/ijms26168028/s1.
